# Simple and efficient germline copy number variant visualization method for the Ion AmpliSeq™ custom panel

**DOI:** 10.1002/mgg3.399

**Published:** 2018-04-06

**Authors:** Shin‐ya Nishio, Hideaki Moteki, Shin‐ichi Usami

**Affiliations:** ^1^ Department of Otorhinolaryngology Shinshu University School of Medicine Matsumoto City Japan

**Keywords:** copy number variation, Ion AmpliSeq, multiplex PCR, next‐generation sequencing, target enrichment

## Abstract

**Background:**

Recent advances in molecular genetic analysis using next‐generation sequencing (NGS) have drastically accelerated the identification of disease‐causing gene mutations. Most next‐generation sequencing analyses of inherited diseases have mainly focused on single‐nucleotide variants and short indels, although, recently, structure variations including copy number variations have come to be considered an important cause of many different diseases. However, only a limited number of tools are available for multiplex PCR‐based target genome enrichment.

**Methods:**

In this paper, we reported a simple and efficient copy number variation visualization method for Ion AmpliSeq™ target resequencing data. Unlike the hybridization capture‐based target genome enrichment system, Ion AmpliSeq™ reads are multiplex PCR products, and each read generated by the same amplicon is quite uniform in length and position. Based on this feature, the depth of coverage information for each amplicon included in the barcode/amplicon coverage matrix file was used for copy number detection analysis. We also performed copy number analysis to investigate the utility of this method through the use of positive controls and a large Japanese hearing loss cohort.

**Results:**

Using this method, we successfully confirmed previously reported copy number loss cases involving the *STRC* gene and copy number gain in trisomy 21 cases. We also performed copy number analysis of a large Japanese hearing loss cohort (2,475 patients) and identified many gene copy number variants. The most prevalent copy number variation was *STRC* gene copy number loss, with 129 patients carrying this copy number variation.

**Conclusion:**

Our copy number visualization method for Ion AmpliSeq™ data can be utilized in efficient copy number analysis for the comparison of a large number of samples. This method is simple and requires only easy calculations using standard spread sheet software.

## 1 INTRODUCTION

Recent advances in molecular genetic analysis using next‐generation sequencing (NGS) have drastically accelerated the identification of disease‐causing gene mutations in a relatively short period. Many forms of clinical genetic testing using this technology have been performed for the precise diagnosis of many inherited diseases. Most next‐generation sequencing analyses of inherited diseases have mainly focused on single‐nucleotide variants (SNVs) and short indels, although, recently, structure variations (SVs) including copy number variations (CNVs) have come to be considered an important cause of many different diseases (Conrad et al., 2010; Zhang, Gu, Hurles, & Lupski, 2009). We identified three Japanese hearing loss patients with *STRC* (OMIM 606440) gene copy loss from the results of hybridization capture‐based target genome enrichment with next‐generation sequencing analysis, and we confirmed this CNV by high‐resolution array comparative genomic hybridization (aCGH) (Moteki et al., 2016).

Copy number analysis software for next‐generation sequencing data employs many different strategies (Zhao, Wang, Wang, Jia, & Zhao, 2013 for review) including paired‐end mapping (Korbel et al., 2007), split‐reads (Ye, Schulz, Long, Apweiler, & Ning, 2009), read depth (Magi, Tattini, Pippucci, Torricelli, & Benelli, 2012; Yoon, Xuan, Makarov, Ye, & Sebat, 2009) and combined methods. Most of these tools were developed for whole genome analysis and/or target genome analysis based on hybridization capture. However, only a limited number of tools are available for multiplex PCR‐based target genome enrichment (Boeva et al., 2014; Demidov, Simakova, Vnuchkova, & Bragin, 2016; Goossens et al., 2009; Grasso et al., 2015; Reinecke, Satya, & DiCarlo, 2015).

Ion AmpliSeq™ is a representative method of multiplex PCR‐based target enrichment widely used in the genetic analysis of germline and somatic mutations. The advantages of Ion AmpliSeq™ are the low DNA input volume, high assay success rate, and relatively easy workflow (Nishio, Hayashi, Watanabe, & Usami, 2015; Tsongalis et al., 2014). The data obtained from Ion AmpliSeq™ differ in nature from those obtained from hybridization capture‐based target genome enrichment. One of the differences is that Ion AmpliSeq data are single‐ended, and pair‐reads or split‐read methods cannot be used for CNV analysis. In addition, Ion AmpliSeq uses multiplex PCR products as sequencing libraries, and most of the target sequence is covered by only one amplicon.

For copy number analysis of Ion AmpliSeq data, Ion Reporter software (ThermoFisher Scientific, MA, USA) is widely used. However, Ion Reporter is mainly designed for case versus control comparisons for CNV detection and is not suitable for a large number of samples requiring high throughput.

In this report, we describe an efficient CNV visualization method for Ion AmpliSeq data, which can be utilized in efficient CNV analysis for the comparison of a large number of samples. This method is simple and requires only easy calculations using standard spread sheet software such as Microsoft Excel. A sample spread sheet is available online (https://nishio470.wixsite.com/examplespreadsheet).

## 2 SUBJECTS AND METHODS

### 2.1 Subjects

A total of 2,475 Japanese patients with bilateral sensorineural hearing loss from 67 ENT departments nationwide participated in this study. Informed written consent was obtained from all subjects, their next of kin, caretakers, or guardians (in the case of minors) prior to participation in the project. This study was approved by the Shinshu University Ethical Committee as well as the ethical committees of each of the other participating institutions.

### 2.2 Amplicon library preparation and sequencing

Amplicon libraries of the target exons were prepared with an Ion AmpliSeq™ Custom Panel (ThermoFisher Scientific, MA, USA) designed using Ion AmpliSeq™ Designer (https://www.ampliseq.com) for 63 or 68 genes (listed in Table S1) (Kitano et al., 2017; Nishio et al., 2015) reported to cause nonsyndromic hearing loss (Hereditary Hearing loss Homepage; http://hereditaryhearingloss.org/) with the Ion AmpliSeq™ Library Kit 2.0 (ThermoFisher Scientific) and Ion Xpress™ Barcode Adapter 1‐96 Kit (ThermoFisher Scientific) according to the manufacturer's instructions. After the amplicon libraries were prepared, equal amounts of the libraries for six patients were pooled for one Ion PGM™ sequence reaction and those for 45 patients were pooled for one Ion Proton™ sequencing.

The emulsion PCR was performed with the Ion OneTouch™ System and Ion OneTouch 200 Template Kit v2 (ThermoFisher Scientific) or Ion PI™ Hi‐Q™ OT2 200 Kit according to the manufacturer's instructions. Sequencing was performed with an Ion torrent PGM™ system using the Ion PGM™ 200 Sequencing Kit and Ion 318™ Chip (ThermoFisher Scientific), or Ion Proton™ system using the Ion PI™ Hi‐Q™ Sequencing 200 Kit and Ion PI™ Chip (ThermoFisher Scientific) according to the manufacturer's instructions.

### 2.3 Base call and data analysis

The sequence data were processed with standard Ion Torrent Suite™ Software ver 5.0 on the Torrent Server and used to successively map the human genome sequence (build GRCh37/hg19) with a Torrent Mapping Alignment Program optimized to Ion Torrent data. After the sequence mapping, the DNA variant regions were piled up with Torrent Variant Caller plug‐in software set to run at high stringency. The depth of coverage information was obtained by Torrent Coverage Analysis plug‐in software (ver. 5.0.4) with custom AmpliSeq™ panel information.

### 2.4 Normalization of depth of coverage data

Unlike the hybridization capture‐based target genome enrichment system, Ion AmpliSeq™ reads were multiplex PCR products, so each read generated by the same amplicon is quite uniform in length and position. Based on this feature of the Ion AmpliSeq™ reads, it was not necessary to count the depth of coverage of each base from BAM files. Instead of counting each base level depth of coverage, amplicon level depth of coverage information was sufficient for further analysis. The barcode/amplicon coverage matrix file in the coverage analysis plug‐in software output contained the depth of coverage information for each amplicon in each sample.

At a glance, the depth of coverage of each amplicon was not very uniform and also differed between samples (Fig. S1). The read depth of coverage of each amplicon was greatly dependent on the PCR amplification efficiency of each primer set, GC‐contents, PCR product length, template concentration, and other factors. However, in general, amplicons efficiently amplified in one sample were also efficiently amplified in other samples. To confirm this, we compared the depth of coverage information of each sample (Fig. S2). As a result, scatter plots of depth of coverage showed two types of correlation in some comparisons. These two types of correlation reflected the different primer pools used in the library preparation. Our custom AmpliSeq library consisted of two primer pools and unequal amounts of the primer pools in the sequencing library resulted in these two types of correlations in the scatter plots. Therefore, normalization should be performed separately for each primer pool. Primer pool information was included in the Ion AmpliSeq™ Designer output “designed.bed” files. Using this primer pool information, the average depth of each primer pool for each sample could be calculated (Equation (1)). In this equation, $\overset{¯}{x}$ indicates the averaged read depths of primer pool 1 and $\overset{¯}{y}$ indicate the averaged read depths of primer pool 2, and *m* and *n* indicate the total number of amplicons included in primer pool 1 and 2, respectively.

The relative value (relative depth) of each amplicon is then calculated by dividing each amplicon read depth by the averaged read depth of the respective primer pool (Equation (2)). In this equation, *r*_*i*_ indicates the relative depth of amplicon *i* included in primer pool 1 and *s*_*j*_ indicates the relative depth of amplicon *j* included in primer pool 2, while *x*_*i*_ is the depth of amplicon *i* included in primer pool 1 and *y*_*j*_ is the depth of amplicon *j* included in primer pool 2. After this process, the relative depth of coverage of the samples is comparable (Fig. S3).

(1) $$\overset{¯}{x}=(x_{1}+x_{2}+\ldots +x_{m})/m$$

$$\overset{¯}{y}=(y_{1}+y_{2}+\ldots +y_{n})/n$$

(2) $$r_{i}=x_{i}/\overset{¯}{x}$$

$$s_{j}=y_{j}/\overset{¯}{y}.$$

To visualize copy number variations, we employed a population‐based comparative approach to reduce the bias among samples and amplicons, and calculated the averaged relative depth of each amplicon for all patients (Equation (3)). In the equation, *r*_*i* all_ indicates the averaged relative read depth of amplicon *i* in primer pool 1 for all patients, and $\overset{¯}{s}$_*j* all_ indicates the averaged relative read depth of amplicon *j* in primer pool 2 for all patients, while *r*_*ip*_ and *s*_*jp*_ indicate the relative read depths of amplicons *i* and *j*, respectively, for patient number *p*. In this study, we employed a total patient number = 45 as an example for further analysis (We analyzed 45 samples by one Ion Proton™ sequencing and we regarded 45 samples as one batch reaction, so we employed data for 45 patients in one CNV analysis) but this can be modified to a more appropriate value if necessary. Normalized relative depth was calculated from the twofold value of the ratio of each relative depth by dividing the average value of the same amplicon by the relative depth of all samples (Equation (4)). These values indicate the relative read depth shift compared to the all patients. In this equation, *t*_*iq*_ and *u*_*jq*_ indicate the normalized relative read depths of amplicons *i* and *j*, respectively, for patient number *q*.

(3) $${\overset{¯}{r}}_{i\;\;\mathrm{all}}=\left(r_{i1}+r_{i2}+\ldots +r_{\mathrm{ip}}\right)/p$$

$${\overset{¯}{s}}_{j\;\;\mathrm{all}}=\left(s_{j1}+s_{j2}+\ldots +s_{\mathrm{jp}}\right)/p$$

(4) $$t_{\mathrm{iq}}=2\times \left(r_{\mathrm{iq}}/{\overset{¯}{r}}_{i\;\;\mathrm{all}}\right)$$

$$u_{\mathrm{jq}}=2\times \left(s_{\mathrm{jq}}/{\overset{¯}{s}}_{j\;\;\mathrm{all}}\right).$$

### 2.5 Removing outliers

After normalization, it is necessary to remove outlier data. Most of the outliers were distributed among the amplicons with lower read depth. As the normalization was performed by calculating the ratio of samples compared to the average for all patients, normalized values are easily affected by differences in depth in lower coverage amplicons (Fig. S4). Based on this distribution, we removed the amplicons with coverage of <0.1 from the averaged relative read depth of all patients (i.e., we removed the amplicons $\overset{¯}{r}$_*i* all_ ≤ 0.1 and $\overset{¯}{s}$_*j* all_ ≤ 0.1).

### 2.6 Clustering and visualization

After removal of outlier data, the normalized relative read depths of amplicons were sorted by chromosome position order and separated by each chromosome and gene. A graph of normalized relative read depths can be drawn using any spread sheet software such as Microsoft Excel. For easy assessment of large copy number changes, smoothing of relative data for five amplicons can also be drawn into the same graph. All CNVs were curated through manual inspection from the graph in this study. We also picked up the amplicons exceeding ±1 standard deviation for all amplicons as CNV candidates. In addition, we employed the *t*‐test to identify statistically different normalized relative read depths for five continuous amplicons compared to the normalized relative read depth of all amplicons to aid the manual inspection. For further statistical copy number variation detection, please refer to other methods using the Hidden Markov Model to estimate the copy number from observed data if necessary (Shen, Gu, & Pe'er, 2001).

## 3 RESULTS AND DISCUSSION

### 3.1 CNV detection in previously reported cases and trisomy 21 cases

**Figure 1:**
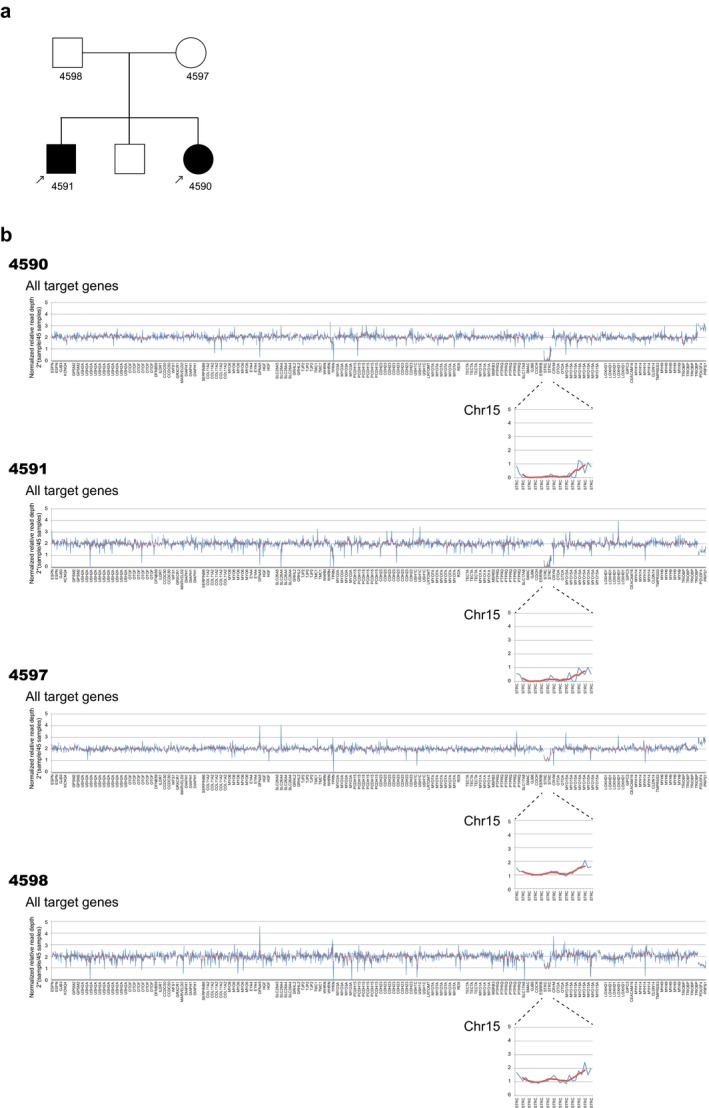
Previously reported Japanese hearing loss patients with *STRC* gene copy loss. (a) Pedigree of a previously reported Japanese hearing loss patient with *STRC* gene copy loss confirmed by high‐resolution array comparative genomic hybridization (aCGH). (b) Copy number variations visualized by our method. As shown in this figure, this method successfully identified the *STRC* gene copy loss variants in both of the two copy loss patients and one copy loss family members. Blue indicates the normalized relative read depth of each amplicon and red indicates the smoothing value for five relative amplicons

To assess the utility of this method, we analyzed three previously reported Japanese hearing loss patients with *STRC* gene copy loss identified from the results of next‐generation sequencing analysis with hybridization capture‐based target genome enrichment, and we confirmed this CNV by high‐resolution array comparative genomic hybridization (aCGH) (Moteki et al., 2016). As a result, we were able to identify the *STRC* gene copy loss variants easily in both of the two copy loss probands and one copy loss family member (Figure 1 shows one of the three cases). These analyses were performed with a 63‐gene AmpliSeq™ custom panel.

**Figure 2:**
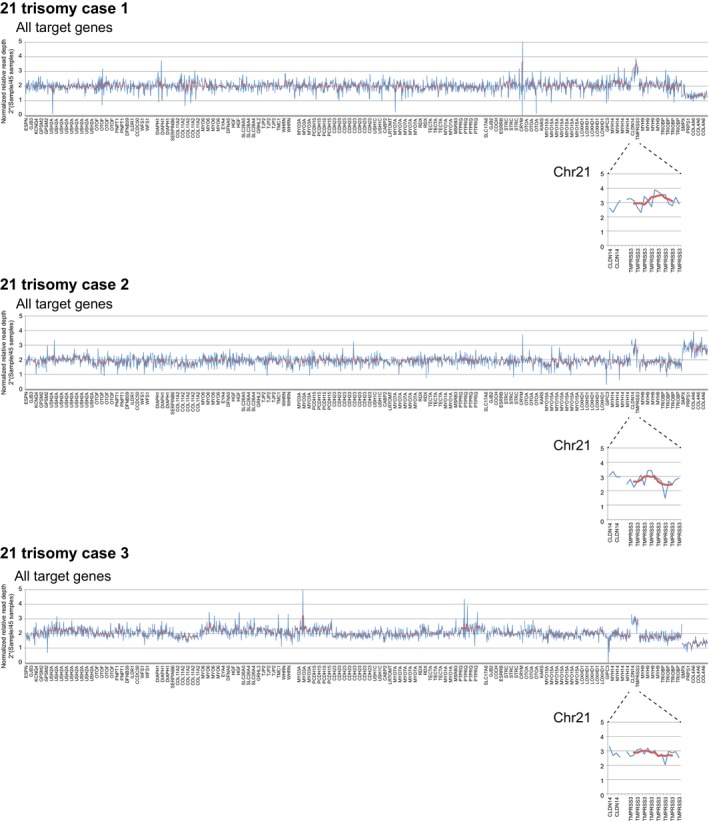
Copy number gain in trisomy 21 cases confirmed by our method. We also analyzed trisomy 21 cases as another positive control. In our Ion AmpliSeq™ custom panel, *CLDN14* and *TMPRSSS3* encoded on the chromosome 21 were included. We were able to identify one copy gains for the *CLDN14* and *TMPRSS3* genes, which reflect a one copy gain of chromosome 21 in all three cases. Blue indicates the normalized relative read depth of each amplicon and red indicates the smoothing value for five relative amplicons

In addition, we also analyzed trisomy 21 cases as a positive control (Figure 2) using a 68‐gene AmpliSeq™ custom panel. In our Ion AmpliSeq™ custom panel, two genes (*CLDN14* (OMIM 605608) and *TMPRSSS3* (OMIM 605511)) encoded on chromosome 21 were included. In all three cases with trisomy 21, we identified 1 copy gains for the *CLDN14* and *TMPRSS3* genes, which reflects a 1 copy gain of chromosome 21. In both the *STRC* and trisomy 21 cases, we could easily detect the copy number loss or copy number gain in cases with different target panels consisting of different kinds of amplicons. It is noteworthy that patient gender in all cases shown in Figures 1 and 2 could easily be distinguished from the copy number of chromosome X genes. In our method, X chromosome genes are not calculated separately, so these differences in the X chromosome are reflected in the value relative to the average of 45 patients. If the gender ratio among 45 patients was close to 1:1, the averaged value for the X chromosome of the 45 patients would have been 1.5 copies. So, the normalized value for X chromosome genes should be close to 2 × (1/1.5) = 1.3 for males, and 2 × (2/1.5) = 2.7 for females.

In addition, both the 63‐gene and 68‐gene Ion AmpliSeq™ custom panels consisting of 2,064 amplicons and 2,010 amplicons, respectively, were compatible with this analytical method, suggesting that this method will be useful for any custom design panels used for other disorders.

To obtain further evidence in support of this CNV detection method, we also performed CNV detection for eight cases without any CNVs in the target genes, as confirmed by aCGH analysis, as negative controls (Fig. S5), and eight cases with *STRC* gene CNVs, one case with an *EYA4* (OMIM 603550) gene CNV, one case with a *POU3F4* (OMIM 300039) gene CNV, one case with an *OTOA* (OMIM 607038) gene CNV, and three cases with trisomy 21, as confirmed by aCGH analysis, as positive controls (Fig. S6).

### 3.2 Copy number variation detection for a large number of Japanese hearing loss patients

Using the above method, we analyzed the target resequencing data for 2,475 Japanese hearing loss patients. Among the 2,475 cases, 104 (4.2%) could not be analyzed due to fluctuations or scattered data. The main reason for these scattered data was particularly large imbalances in read depth between primer pool 1 and 2. We measured the final library concentration obtained from primer pool 1 and 2 using a TaqMan library quantitation kit and the Step One Plus real‐time PCR system; however, this quantitation was not accurate in some cases. The most plausible reason for this inaccurate quantification was the effect of primer dimers. This phenomenon can be improved by adding an extra one‐time Ampure XP purification, a different method of library quantitation using a Qubit fluorometer or Bioanalyzer, or using an equalizer kit for library preparation.

Among the 2,371 patients for whom data were successfully analyzed, 234 patients had some CNVs (Table 1 and details of the deduced CNV positions are given in Table S2). *STRC* gene copy number loss was the most prevalent CNV, with 129 patients having a copy number loss mutation of this gene (88 cases with a one copy loss and 41 cases with a two copy loss). *STRC* gene copy number gain mutation was the second most prevalent CNV, with 55 patients carrying a *STRC* gene copy number gain (53 patients carried a one copy number gain and 2 patients carried a two copy gain); however, the clinical impact of *STRC* gene copy number gain mutations is unknown, although it is presumably neutral or benign. As for other genes, we identified various gene CNVs in the *CABP2* (OMIM 607314)*, CDH23* (OMIM 605516)*, CLDN14, CLDN14/TMPRSS3, ESPN* (OMIM 606351)*, EYA4, GIPC3* (OMIM 608792)*, GPSM2* (OMIM 609245)*, MYO15A* (OMIM 602666)*, MYO3A* (OMIM 606808)*, OTOA, PCDH15* (OMIM 605514)*, PNPT1* (OMIM 610316)*, POU3F4, SERPINB6* (OMIM 173321)*, SLC17A8* (OMIM 607557)*, STRC/OTOA, TMC1* (OMIM 606706)*, TMPRSS3,* and *WFS1* (OMIM 606201) genes. However, prior to the clinical application of this method, all CNVs identified using next‐generation sequencing should be confirmed by orthogonal methods using different measurement modalities such as aCGH. In addition, family segregation analysis should be performed to classify appropriately the pathogenicity of the identified CNVs. Some of the CNVs identified in this study will be “benign” or “neutral” and will not have any clinical impact. We are currently performing aCGH analysis to confirm the CNVs detected by next‐generation sequencing analysis with the reported method and also undertaking segregation analysis using the family members.

**Table 1:** Copy number variations identified in a large Japanese hearing loss cohort using our method

Gene	CNV		No. of patients
*CABP2*	1	Copy gain	2
*CDH23*	1	Copy loss	1
*CLDN14*	1	Copy gain	1
*CLDN14/TMPRSS3*	1	Copy gain	6
*ESPN*	1	Copy gain	1
*EYA4*	1	Copy loss	2
*GIPC3*	1	Copy loss	2
*GPSM2*	1	Copy gain	1
*MYO15A*	1	Copy gain	1
*MYO15A*	1	Copy loss	8
*MYO3A*	1	Copy gain	1
*OTOA*	1	Copy gain	8
*OTOA*	1	Copy loss	5
*PCDH15*	1	Copy loss	1
*PNPT1*	1	Copy loss	1
*POU3F4*	1	Copy loss	2
*SERPINB6*	1	Copy loss	1
*SLC17A8*	1	Copy gain	1
*STRC*	1	Copy gain	53
*STRC*	2	Copy gain	2
*STRC*	1	Copy loss	88
*STRC*	2	Copy loss	41
*STRC/OTOA*	1	Copy gain	1
*STRC/OTOA*	1	Copy loss	1
*TMC1*	1	Copy gain	1
*TMPRSS3*	1	Copy loss	1
*WFS1*	2	Copy gain	1

### 3.3 Case study

**Figure 3:**
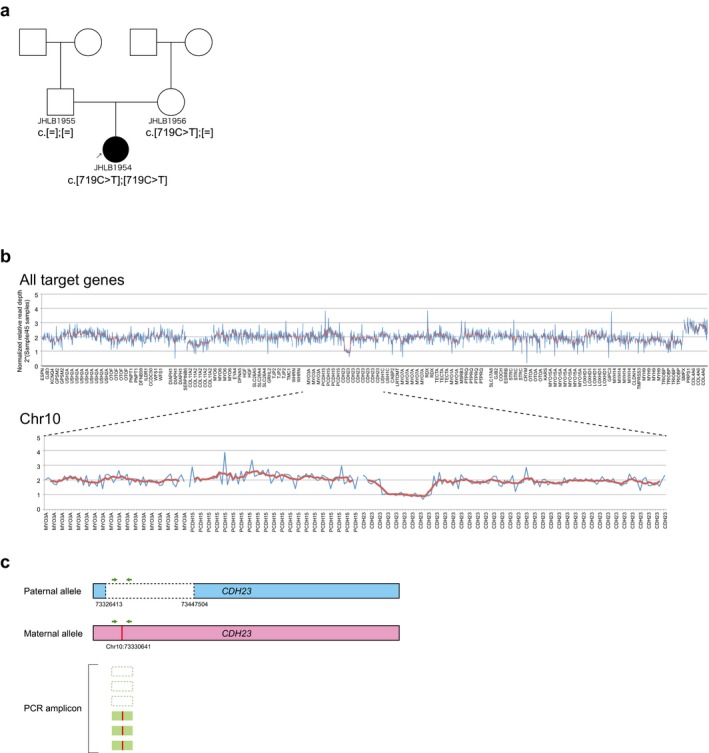
A *CDH23* copy number loss case identified in this study. (a) Pedigree and identified variant. Target resequencing analysis results for this patient showed a homozygous *CDH23:NM_022124.5:c.719C>T:p.P240L* mutation. (b) Results of CNV analysis. This patient carried a one copy loss mutation of the *CDH23* gene. Blue indicates the normalized relative read depth of each amplicon and red indicates the smoothing value for five relative amplicons. (c) Schema of the pseudohomozygosity for this patient. The pseudohomozygous *c.719C>T* mutation was caused by an amplicon from the mutated allele without any CNV in this patient. Therefore, the true genetic cause of the hearing loss in this patient was a compound heterozygous mutation of *CDH23:NM_022124.5:c.719C>T:p.P240L* mutation with a one copy number loss mutation

As an example case study, we herein describe a case with *CDH23* gene copy number loss (Figure 3). This case carried a one copy number loss mutation in the *CDH23* gene ranging from position 73,326,413 to 73,447,504 on chromosome 10 (build hg19/GRCh37). In our target resequencing analysis results, we identified a homozygous *CDH23:NM_022124.5:c.719C>T:p.P240L* mutation in this patient and we regarded this homozygous mutation to be the genetic cause of hearing loss in this patient. However, this patient carried a one copy number loss mutation of the *CDH23* gene so that the homozygous *CDH23:NM_022124.5:c.719C>T:p.P240L* mutation identified in this patient should be genotyping error (Figure 3c). Therefore, the genetic cause of deafness in this case was eventually concluded to be a compound heterozygous mutation of *CDH23:NM_022124.5:c.719C>T:p.P240L* mutation with a one copy number loss mutation.

## 4 CONCLUSION

In this paper, we reported a simple and efficient copy number variation visualization method for Ion AmpliSeq™ data. Using this method, we successfully confirmed the copy number loss of the *STRC* gene and copy number gain of trisomy 21 cases as positive controls. These cases were analyzed using different primer sets, so this method is thought to be compatible with other custom design panels for use with other disorders. We also performed copy number analysis for a large Japanese hearing loss cohort and identified many gene copy number variants. Further analysis is required to confirm the copy number variants and their pathogenicity classification.

## CONFLICT OF INTEREST

None of the authors have any competing financial interests to declare.
