# Supplementary figures and images for: Simple and efficient germline copy number variant visualization method for the Ion AmpliSeq™ custom panel
Source: Mol Genet Genomic Med. 2018 Apr 6;6(4):678–86. doi: 10.1002/mgg3.399 (PMC6081219; doi:10.1002/mgg3.399)

Sample #01

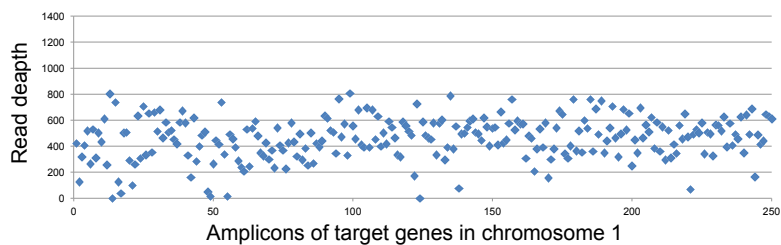

Sample #02

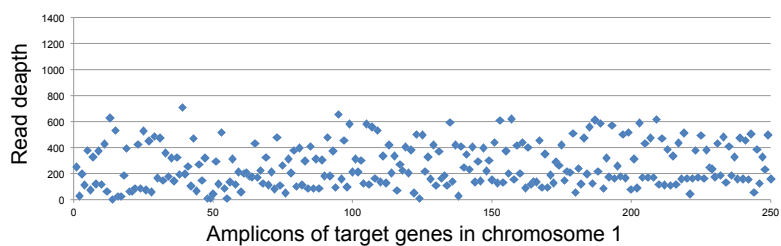

Sample #03

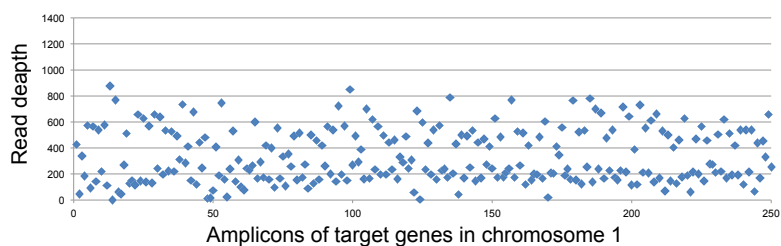

Sample #04

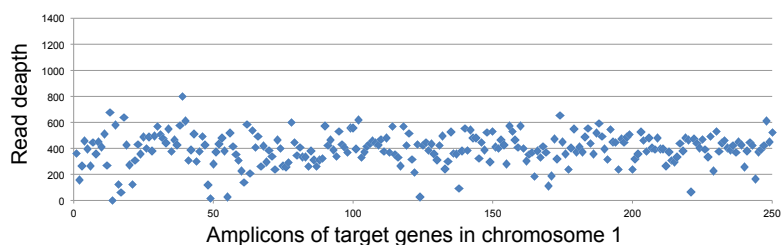

Sample #05

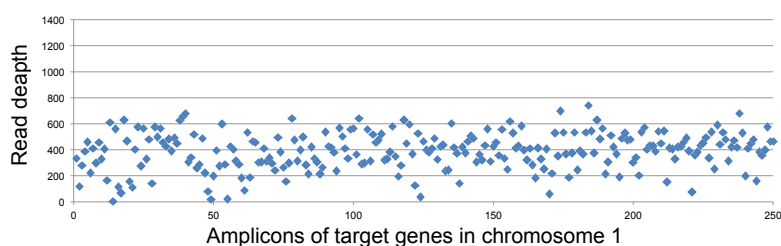

Sample #06

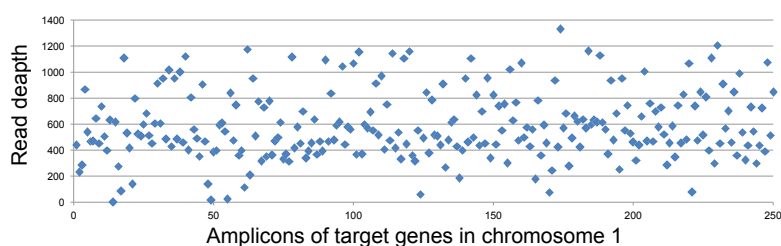

Sample #07

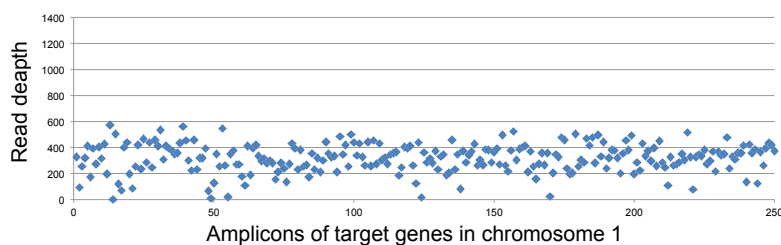

Sample #08

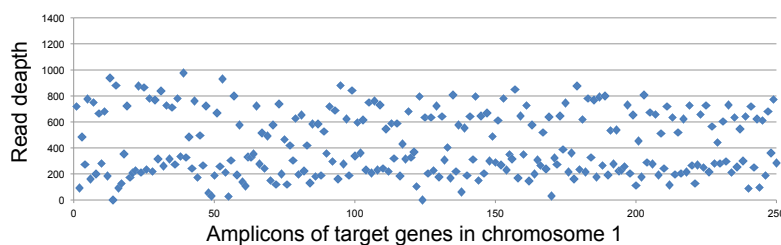

Supplement: Supplementary file 1 [file MGG3-6-678-s001.pdf]

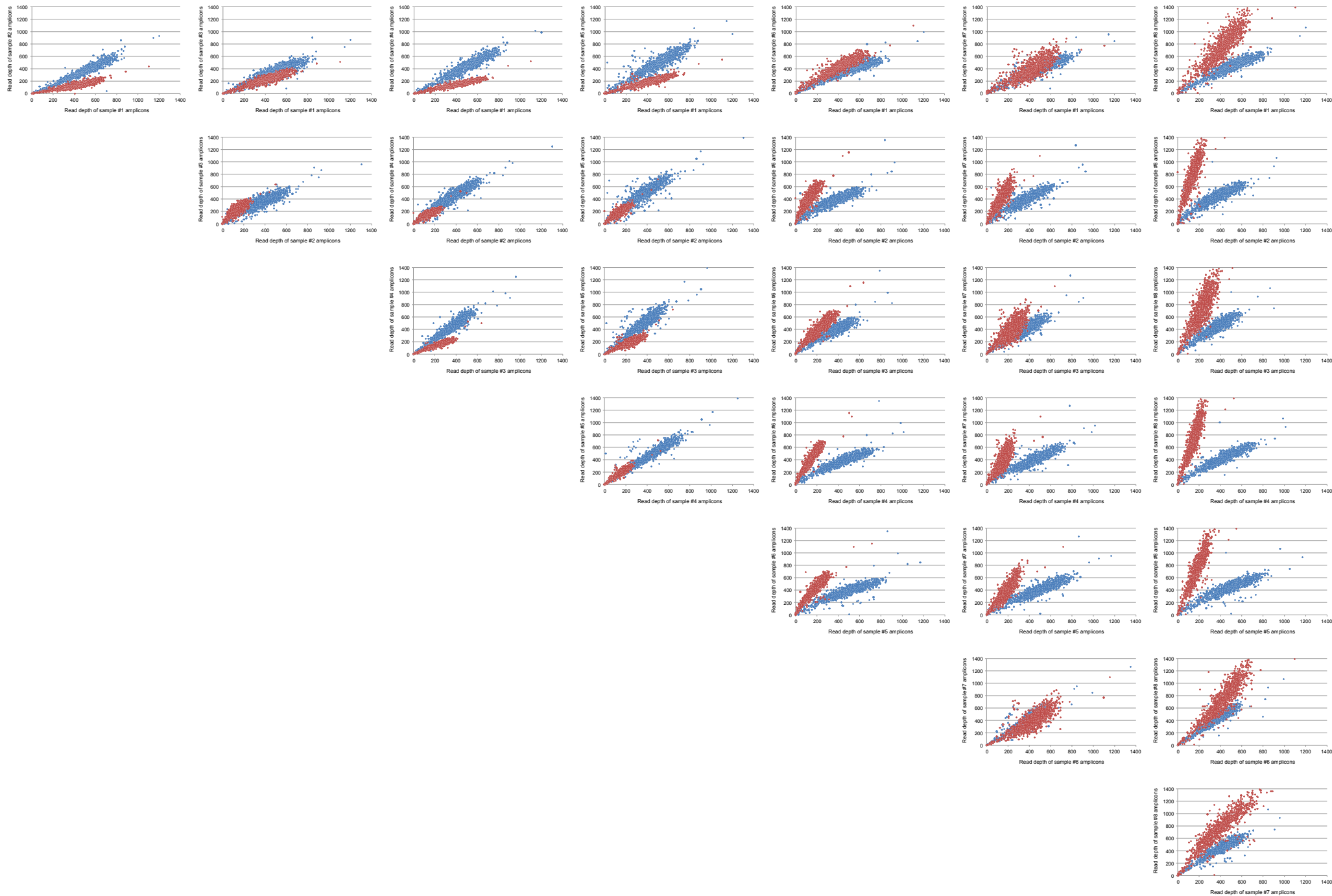

Supplement: Supplementary file 2 [file MGG3-6-678-s002.pdf]

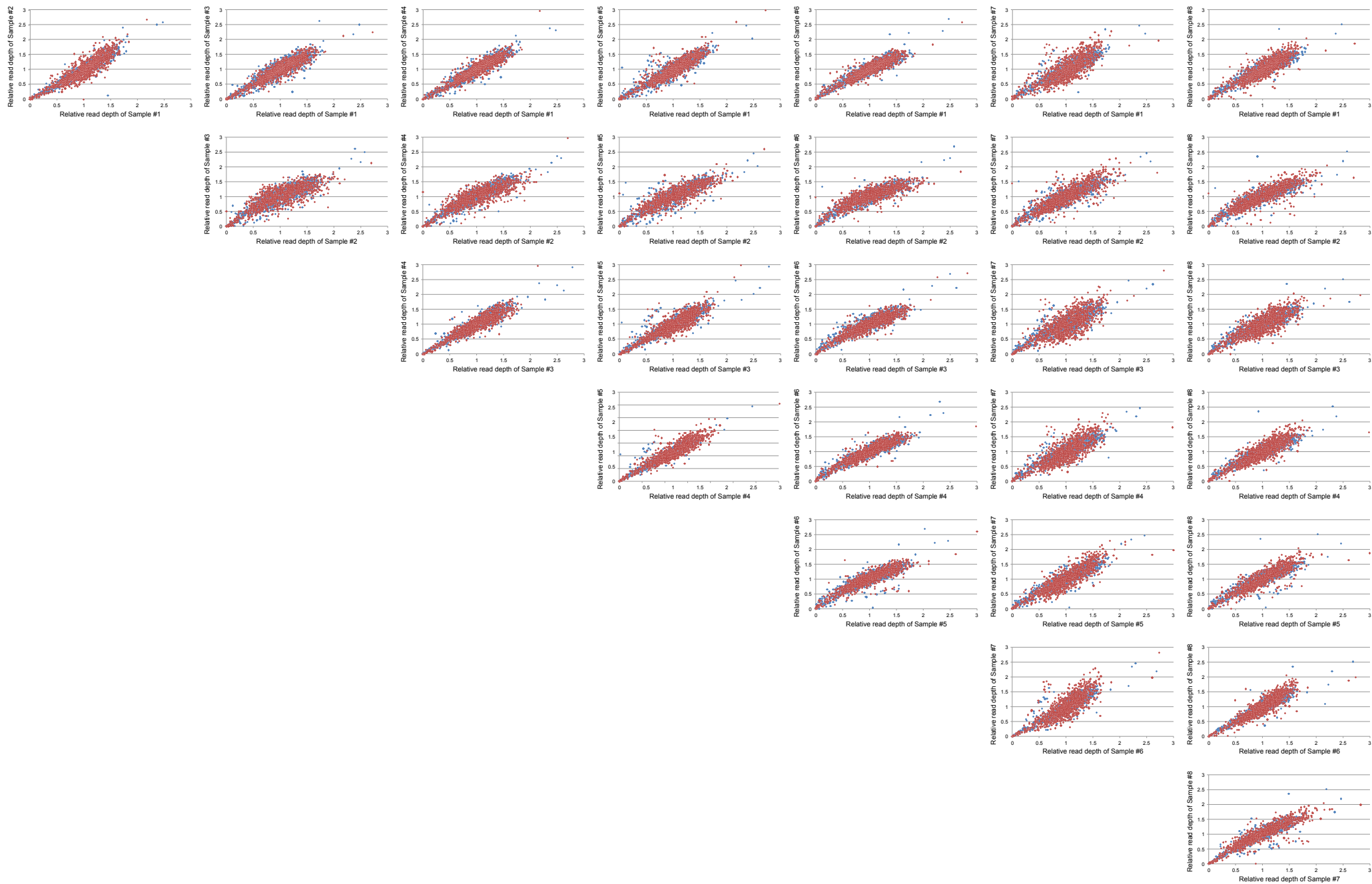

Supplement: Supplementary file 3 [file MGG3-6-678-s003.pdf]

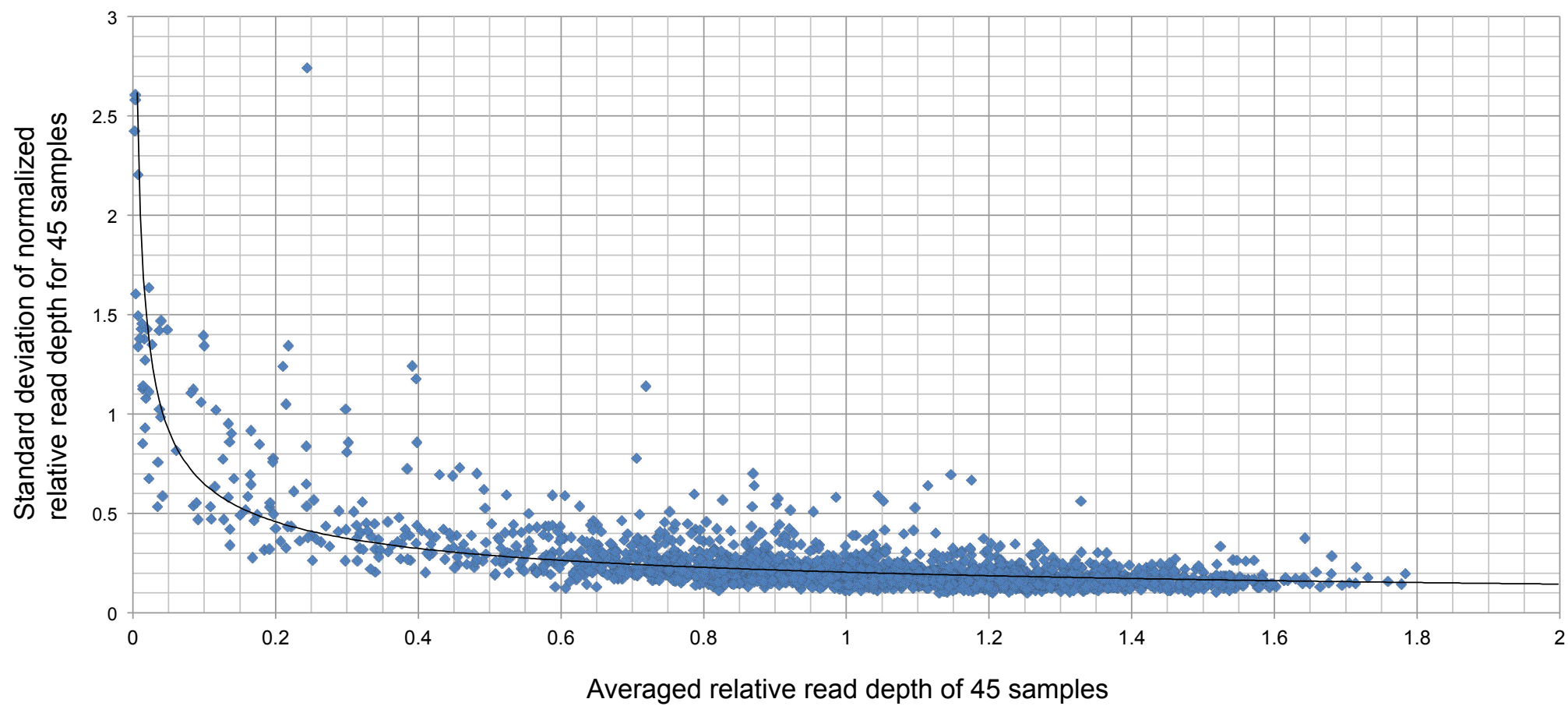

Supplement: Supplementary file 4 [file MGG3-6-678-s004.pdf]
