# Supplementary material for: Simple and efficient germline copy number variant visualization method for the Ion AmpliSeq™ custom panel
Source: Mol Genet Genomic Med. 2018 Apr 6;6(4):678–86. doi: 10.1002/mgg3.399 (PMC6081219; doi:10.1002/mgg3.399)

aCGH confirmed negative control case #1

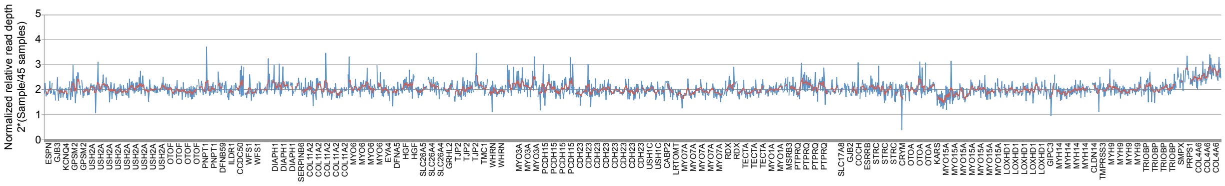

aCGH confirmed negative control case #2

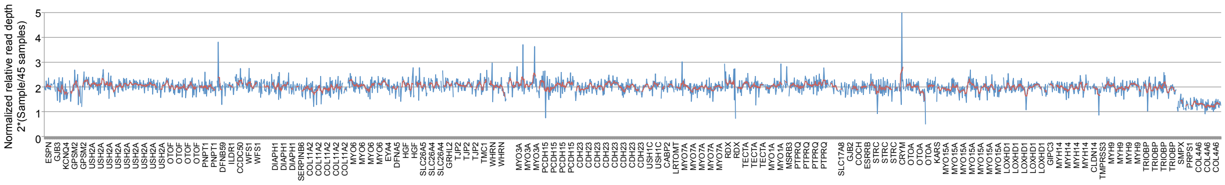

aCGH confirmed negative control case #3

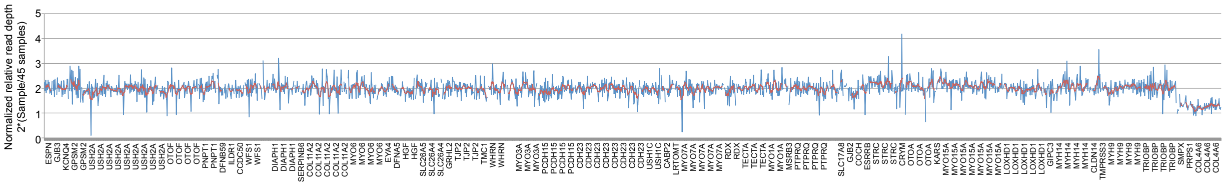

aCGH confirmed negative control case #4

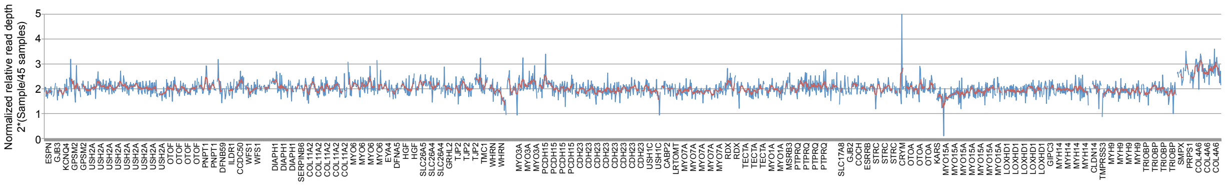

aCGH confirmed negative control case #5

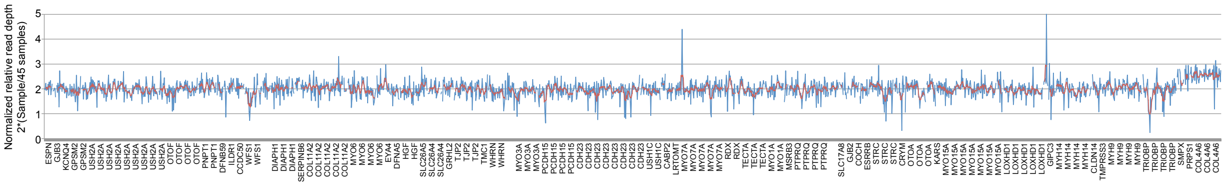

aCGH confirmed negative control case #6

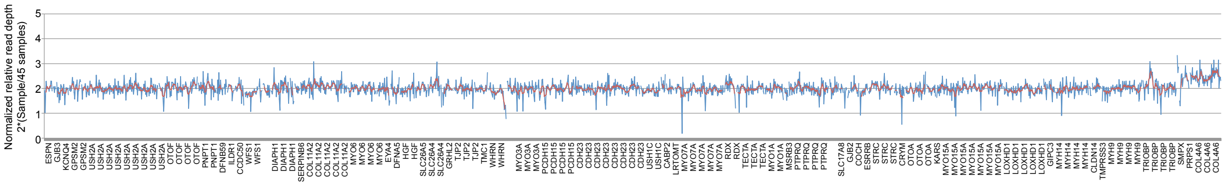

aCGH confirmed negative control case #7

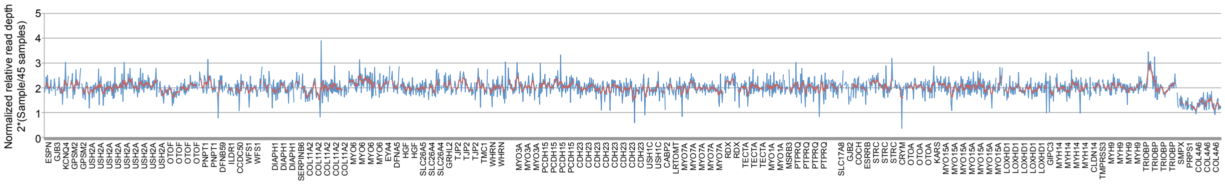

aCGH confirmed negative control case #8

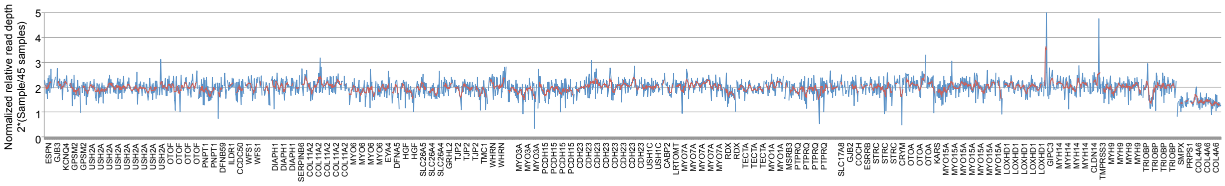

Supplement: Supplementary file 5 [file MGG3-6-678-s005.pdf]
