# Supplementary material for: Simple and efficient germline copy number variant visualization method for the Ion AmpliSeq™ custom panel
Source: Mol Genet Genomic Med. 2018 Apr 6;6(4):678–86. doi: 10.1002/mgg3.399 (PMC6081219; doi:10.1002/mgg3.399)

aCGH confirmed positive control case #1 (*STRC* two copy loss case)

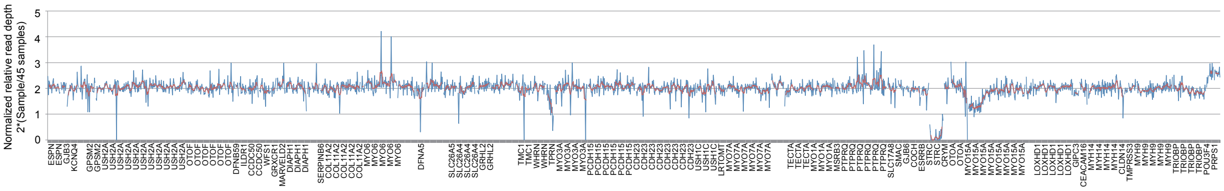

aCGH confirmed positive control case #2 (*STRC* two copy loss case)

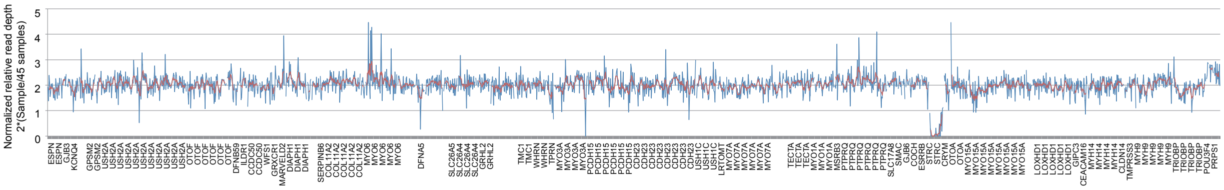

aCGH confirmed positive control case #3 (*STRC* two copy loss case)

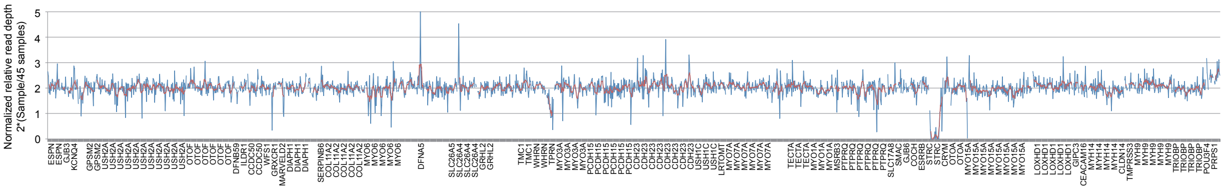

aCGH confirmed positive control case #4 (*STRC* one copy loss case)

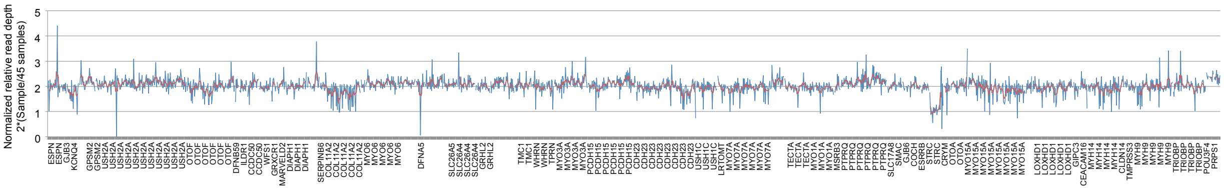

aCGH confirmed positive control case #5 (*STRC* one copy loss case)

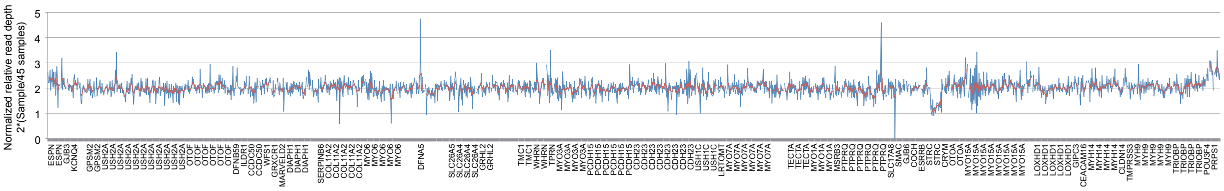

aCGH confirmed positive control case #6 (*STRC* one copy loss case)

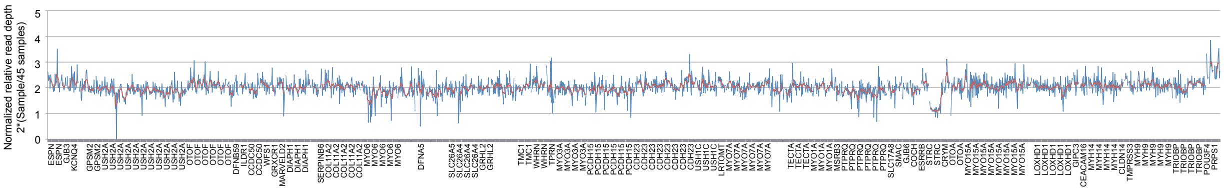

aCGH confirmed positive control case #7 (*STRC* one copy gain case)

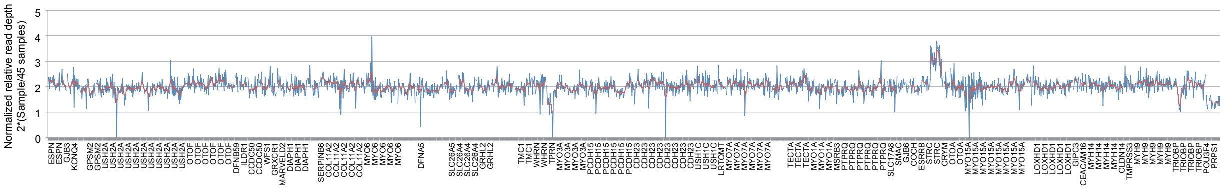

aCGH confirmed positive control case #8 (*STRC* one copy gain case)

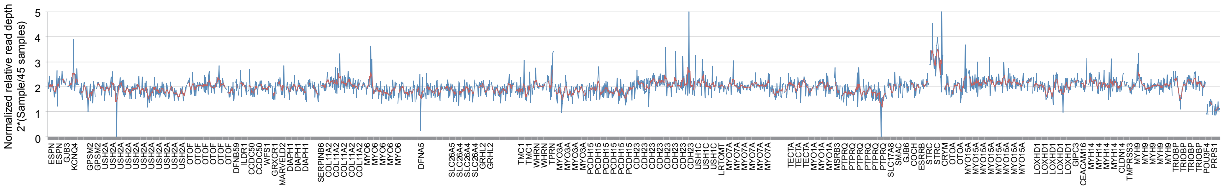

aCGH confirmed positive control case #9 (*EYA4* one copy loss case)

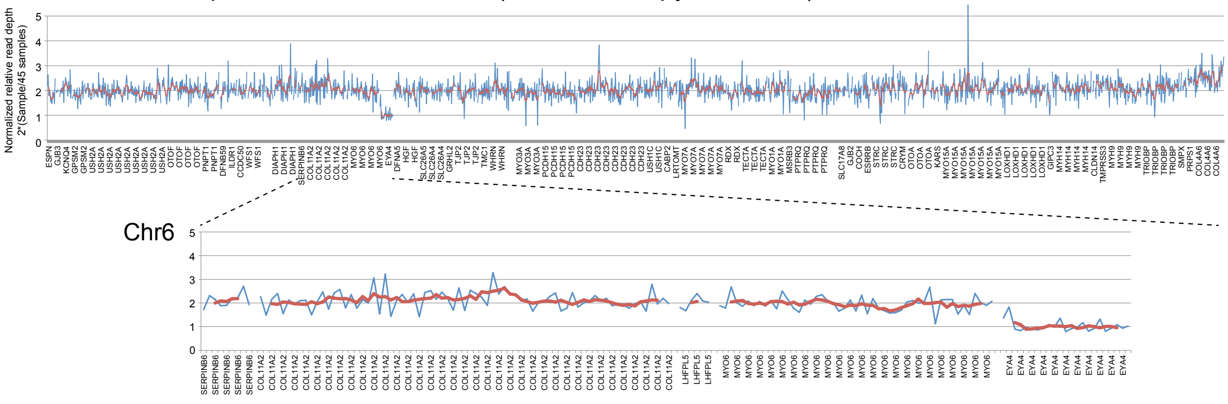

aCGH confirmed positive control case #10 (*POU3F4* one copy loss case)

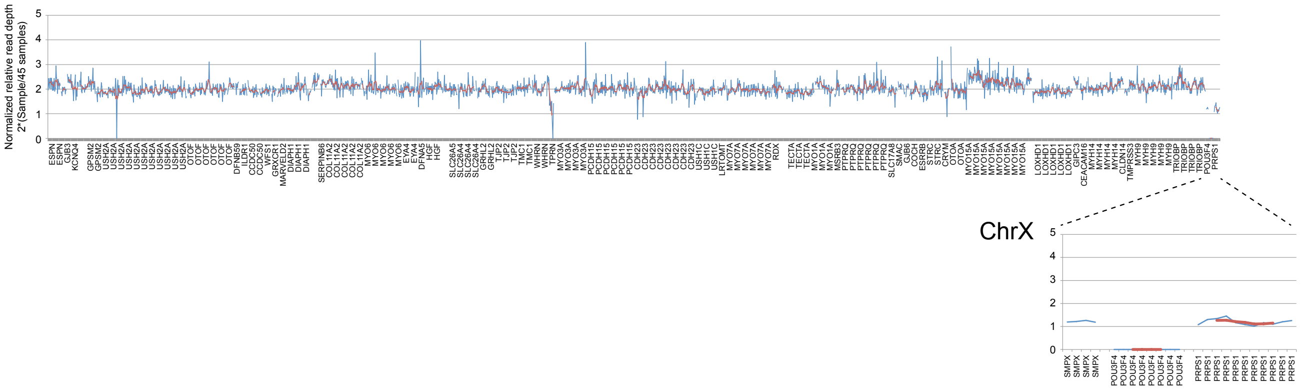

aCGH confirmed positive control case #11 (*OTOA* one copy gain case)

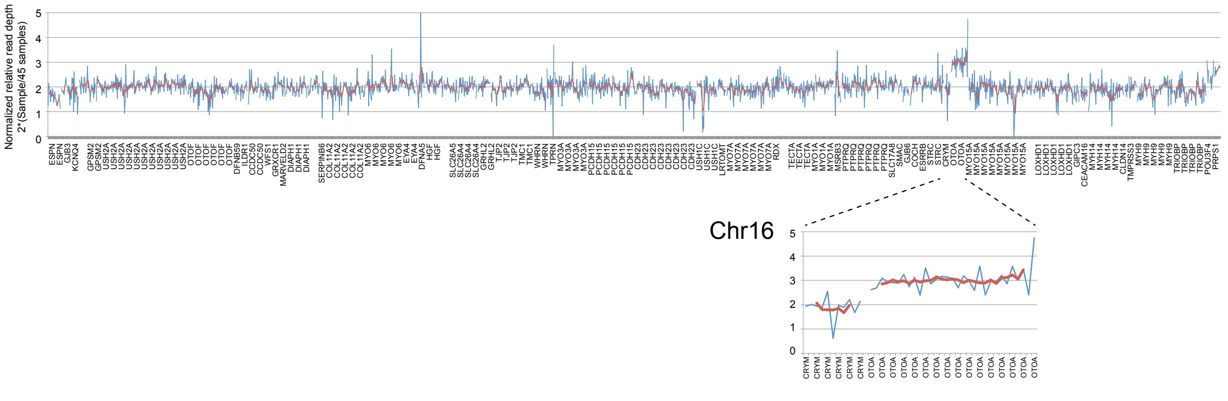

aCGH confirmed positive control case #12 (Trisomy 21 case)

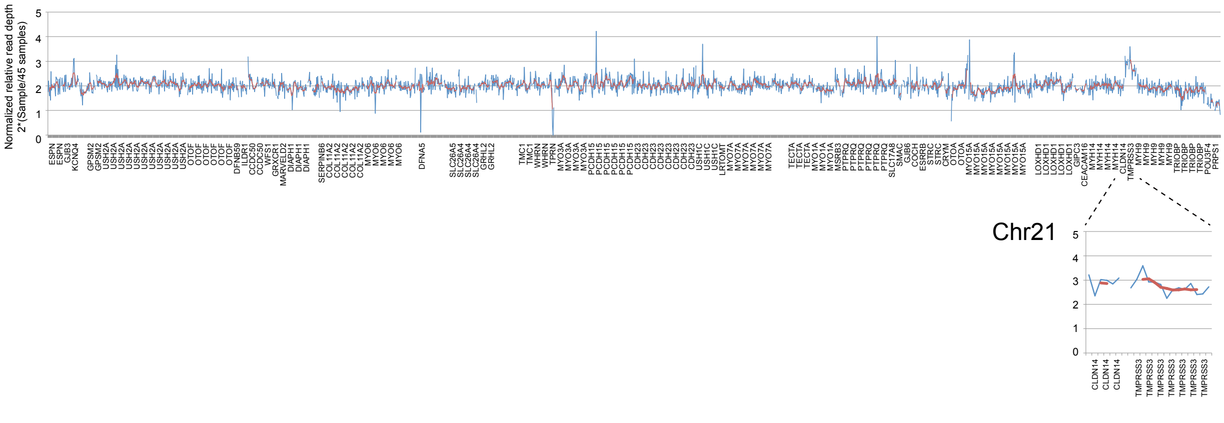

aCGH confirmed positive control case #13 (Trisomy 21 case)

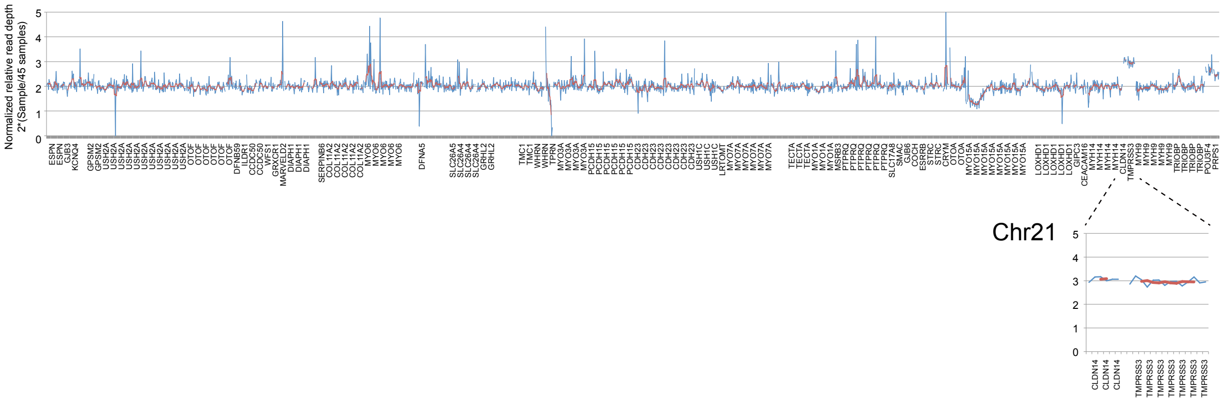

aCGH confirmed positive control case #14 (Trisomy 21 case)

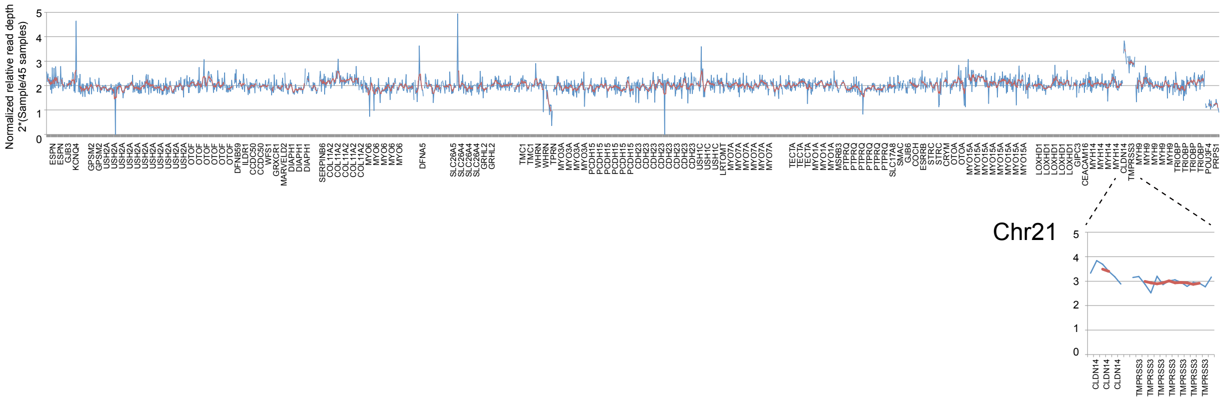

Supplement: Supplementary file 6 [file MGG3-6-678-s006.pdf]
