# Supplementary material for: Simple and efficient germline copy number variant visualization method for the Ion AmpliSeq™ custom panel
Source: Mol Genet Genomic Med. 2018 Apr 6;6(4):678–86. doi: 10.1002/mgg3.399 (PMC6081219; doi:10.1002/mgg3.399)
Supplement: Supplementary file 7 [file MGG3-6-678-s007.pdf]

Deafness pannel v2

1	ACTG1
2	CCDC50
3	CDH23
4	CEACAM16
5	CLDN14
6	COCH
7	COL11A2
8	CRYM
9	DFNA5
10	DFNB59
11	DIAPH1
12	ESPN
13	ESRRB
14	EYA4
15	GIPC3
16	GJB2
17	GJB3
18	GJB6
19	GPSM2
20	GRHL2
21	GRXCR1
22	HGF
23	ILDR1
24	KCNQ4
25	LHFPL5
26	LOXHD1
27	LRTOMT
28	MARVELD2
29	MIRN96
30	MSRB3
31	MYH14
32	MYH9
33	MYO15A
34	MYO1A
35	MYO3A
36	MYO6
37	MYO7A
38	OTOA
39	OTOF
40	PCDH15
41	POU3F4
42	POU4F3
43	PRPS1
44	PTPRQ
45	RDX
46	SERPINB6
47	SLC17A8
48	SLC26A4
49	SLC26A5
50	SMAC
51	SMPX
52	STRC
53	TECTA
54	TJP2
55	TMC1
56	TMIE
57	TMPRSS3
58	TPRN
59	TRIOBP
60	USH1C
61	USH2A
62	WFS1
63	WHRN

Deafness pannel v3

1	ACTG1
2	CABP2
3	CCDC50
4	CDH23
5	CEACAM16
6	CIB2
7	CLDN14
8	COCH
9	COL11A2
10	COL4A6
11	CRYM
12	DFNA5
13	DFNB59
14	DIAPH1
15	ESPN
16	ESRRB
17	EYA4
18	GIPC3
19	GJB2
20	GJB3
21	GJB6
22	GPSM2
23	GRHL2
24	GRXCR1
25	HGF
26	ILDR1
27	KARS
28	KCNQ4
29	LHFPL5
30	LOXHD1
31	LRTOMT
32	MARVELD2
33	MIRN96
34	MSRB3
35	MYH14
36	MYH9
37	MYO15A
38	MYO1A
39	MYO3A
40	MYO6
41	MYO7A
42	OTOA
43	OTOF
44	PCDH15
45	PNPT1
46	POU3F4
47	POU4F3
48	PRPS1
49	PTPRQ
50	RDX
51	SERPINB6
52	SLC17A8
53	SLC26A4
54	SLC26A5
55	SMAC
56	SMPX
57	STRC
58	TECTA
59	TJP2
60	TMC1
61	TMIE
62	TMPRSS3
63	TPRN
64	TRIOBP
65	USH1C
66	USH2A
67	WFS1
68	WHRN
