# Supplementary material for: Simple and efficient germline copy number variant visualization method for the Ion AmpliSeq™ custom panel
Source: Mol Genet Genomic Med. 2018 Apr 6;6(4):678–86. doi: 10.1002/mgg3.399 (PMC6081219; doi:10.1002/mgg3.399)
Supplement: Supplementary file 8 [file MGG3-6-678-s008.pdf]

Gene	CNV_NUM		Deduced CNV position	Number of Case
CABP2	1	copy gain	GRCh37/hg19 11q13.2 (67286457_67290898)x3	1 case
CABP2	1	copy gain	GRCh37/hg19 11q13.2 (67287214_67290898)x3	1 case
CDH23	1	copy loss	GRCh37/hg19 10q22.1 (73326413_73447504)x1	1 case
CLDN14	1	copy gain	GRCh37/hg19 21q22.13 (37833356_37834097)x3	1 case
CLDN14_TMPRSS3	1	copy gain	GRCh37/hg19 21q22.13 (37833223_37833924)x3, GRCh37/hg19 21q22.3 (43792704_43815585)x3	6 cases
ESPN	1	copy gain	GRCh37/hg19 1p36.31 (6488227_6520192)x3	1 case
EYA4	1	copy loss	GRCh37/hg19 6q23.2 (133595855_133849987)x1	1 case
EYA4	1	copy loss	GRCh37/hg19 6q23.2 (133782240_133789915)x1	1 case
GIPC3	1	copy loss	GRCh37/hg19 19p13.3 (3585763_3590265)x1	2 cases
GPSM2	1	copy gain	GRCh37/hg19 8q22.3 (109428059_109472579)x3	1 case
MYO15A	1	copy gain	GRCh37/hg19 17p11.2 (18022064_18055525)x3	1 case
MYO15A	1	copy loss	GRCh37/hg19 17p11.2 (18022064_18025750)x1	1 case
MYO15A	1	copy loss	GRCh37/hg19 17p11.2 (18022064_18029857)x1	1 case
MYO15A	1	copy loss	GRCh37/hg19 17p11.2 (18022064_18034896)x1	1 case
MYO15A	1	copy loss	GRCh37/hg19 17p11.2 (18022064_18035957)x1	1 case
MYO15A	1	copy loss	GRCh37/hg19 17p11.2 (18022070_18034896)x1	2 cases
MYO15A	1	copy loss	GRCh37/hg19 17p11.2 (18022373_18027907)x1	1 case
MYO15A	1	copy loss	GRCh37/hg19 17p11.2 (18022373_18030527)x1	1 case
MYO3A	1	copy gain	GRCh37/hg19 10p12.1 (26409562_26482249)x3	1 case
OTOA	1	copy gain	GRCh37/hg19 16p12.2 (21689705_21771867)x3	1 case
OTOA	1	copy gain	GRCh37/hg19 16p12.2 (21689730_21703065)x3	2 cases
OTOA	1	copy gain	GRCh37/hg19 16p12.2 (21689730_21747745)x3	1 case
OTOA	1	copy gain	GRCh37/hg19 16p12.2 (21689730_21768683)x3	1 case
OTOA	1	copy gain	GRCh37/hg19 16p12.2 (21742087_21768683)x3	2 cases
OTOA	1	copy gain	GRCh37/hg19 16p12.2 (21742087_21771913)x3	1 case
OTOA	1	copy loss	GRCh37/hg19 16p12.2 (21689705_21771867)x1	2 cases
OTOA	1	copy loss	GRCh37/hg19 16p12.2 (21689730_21739828)x1	1 case
OTOA	1	copy loss	GRCh37/hg19 16p12.2 (21689730_21771913)x1	1 case
OTOA	1	copy loss	GRCh37/hg19 16p12.2 (21742087_21768683)x1	1 case
PCDH15	1	copy loss	GRCh37/hg19 10q21.1 (55566302_55571437)x1	1 case
PNPT1	1	copy loss	GRCh37/hg19 2p16.1 (55882012_55914875)x1	1 case
POU3F4	1	copy loss	GRCh37/hg19 Xq21.1 (82763279_82764415)x0	1 case
POU3F4	1	copy loss	GRCh37/hg19 Xq21.1 (82763285_82764461)x1	1 case
SERPINB6	1	copy loss	GRCh37/hg19 6p25.2 (2948486_2959598)x1	1 case
SLC17A8	1	copy gain	GRCh37/hg19 12q23.1 (100751107_100797958)x3	1 case
STRC	1	copy gain	GRCh37/hg19 15q15.3 (43891797_43903244)x3	2 cases
STRC	1	copy gain	GRCh37/hg19 15q15.3 (43891797_43911020)x3	29 cases
STRC	1	copy gain	GRCh37/hg19 15q15.3 (43892127_43895618)x3	1 case
STRC	1	copy gain	GRCh37/hg19 15q15.3 (43892127_43897620)x3	2 cases
STRC	1	copy gain	GRCh37/hg19 15q15.3 (43892127_43906737)x3	8 cases
STRC	1	copy gain	GRCh37/hg19 15q15.3 (43892654_43896940)x3	1 case
STRC	1	copy gain	GRCh37/hg19 15q15.3 (43892690_43897644)x3	1 case
STRC	1	copy gain	GRCh37/hg19 15q15.3 (43892690_43901566)x3	2 cases
STRC	1	copy gain	GRCh37/hg19 15q15.3 (43892690_43903244)x3	2 cases
STRC	1	copy gain	GRCh37/hg19 15q15.3 (43897440_43906737)x3	1 case
STRC	1	copy gain	GRCh37/hg19 15q15.3 (43900006_43903230)x3	1 case
STRC	1	copy gain	GRCh37/hg19 15q15.3 (43900006_43906737)x3	1 case
STRC	1	copy gain	GRCh37/hg19 15q15.3 (43902462_43911020)x3	1 case
STRC	1	copy gain	GRCh37/hg19 15q15.3 (43903015_43911020)x3	1 case
STRC	2	copy gain	GRCh37/hg19 15q15.3 (43892127_43906737)x4	1 case
STRC	2	copy gain	GRCh37/hg19 15q15.3 (43892690_43897644)x4	1 case
STRC	1	copy loss	GRCh37/hg19 15q15.3 (43891797_43900450)x1	3 cases
STRC	1	copy loss	GRCh37/hg19 15q15.3 (43891797_43911020)x1	33 cases
STRC	1	copy loss	GRCh37/hg19 15q15.3 (43892127_43906737)x1	18 cases
STRC	1	copy loss	GRCh37/hg19 15q15.3 (43892690_43897644)x1	1 case
STRC	1	copy loss	GRCh37/hg19 15q15.3 (43897033_43901591)x1	1 case
STRC	1	copy loss	GRCh37/hg19 15q15.3 (43897440_43903230)x1	1 case
STRC	1	copy loss	GRCh37/hg19 15q15.3 (43897440_43903845)x1	1 case
STRC	1	copy loss	GRCh37/hg19 15q15.3 (43897440_43906737)x1	7 cases
STRC	1	copy loss	GRCh37/hg19 15q15.3 (43900006_43901591)x1	1 case
STRC	1	copy loss	GRCh37/hg19 15q15.3 (43900006_43903230)x1	5 cases
STRC	1	copy loss	GRCh37/hg19 15q15.3 (43900006_43903845)x1	2 cases
STRC	1	copy loss	GRCh37/hg19 15q15.3 (43900006_43906737)x1	12 cases
STRC	1	copy loss	GRCh37/hg19 15q15.3 (43902462_43911020)x1	1 case
STRC	2	copy loss	GRCh37/hg19 15q15.3 (43891797_43911020)x0	29 cases
STRC	2	copy loss	GRCh37/hg19 15q15.3 (43892127_43906737)x0	8 cases
STRC	2	copy loss	GRCh37/hg19 15q15.3 (43892690_43901566)x0	1 case
STRC	2	copy loss	GRCh37/hg19 15q15.3 (43892690_43903244)x0	1 case
STRC	2	copy loss	GRCh37/hg19 15q15.3 (43900006_43906737)x0	3 cases
STRC/OTOA	1	copy gain	GRCh37/hg19 15q15.3 (43892127_43897620)x3, GRCh37/hg19 16p12.2 (21689851_21771867)x3	1 case
STRC/OTOA	1	copy loss	GRCh37/hg19 15q15.3 (43900006_43906737)x1, GRCh37/hg19 16p12.2 (21689705_21771867)x1	1 case
TMC1	1	copy gain	GRCh37/hg19 9q21.13 (75263497_75450990)x3	1 case
TMPRSS3	1	copy loss	GRCh37/hg19 21q22.3 (43792704_43815585)x1	1 case
WFS1	2	copy gain	GRCh37/hg19 4p16.1 (6302584_6303681)x4	1 case
